# A new and promising C-phycocyanin-producing cyanobacterial strain, *Cyanobium* sp. MMK01: practical strategy towards developing a methodology to achieve C-phycocyanin with ultra-high purity

**DOI:** 10.3389/fmicb.2024.1394617

**Published:** 2024-11-21

**Authors:** Melika Shafiei, Maral Shafiei, Naeema Mohseni Sani, Wangbiao Guo, Shuaiqi Guo, Hojatollah Vali, Kambiz Akbari Noghabi

**Affiliations:** ^1^Department of Energy and Environmental Biotechnology, National Institute of Genetic Engineering and Biotechnology (NIGEB), Tehran, Iran; ^2^School of Medicine, Yale University, West Haven, CT, United States; ^3^Department of Anatomy & Cell Biology, McGill University, Montreal, QC, Canada

**Keywords:** *Cyanobium* sp. MMK01, C-phycocyanin, ion-exchange chromatography, human lung cancer cells (Calu-6), MTT assay

## Abstract

Selecting a suitable cyanobacterial strain and developing easy-to-afford purification processes are two crucial aspects impacting the optimal production yield and appropriate purity of C-phycocyanin (C-PC). *Cyanobium* sp. MMK01, a highly efficient C-PC-producing bacterium, was identified among four cyanobacterial isolates using morphological characteristics and 16S rRNA gene sequencing. The purification process of C-PC began with ammonium sulfate precipitation, leading to a purity index (PI) of 4.04. Subsequent purification through ion exchange chromatography ultimately resulted in an ultra-highly purified form of C-PC with a significant PI of 5.82. SDS-PAGE analysis of purified C-PC showed the presence of two distinct bands, *α* (13 kDa) and *β* (15 kDa). Significantly effective at scavenging free radicals, C-PC also inhibits the viability of human lung cancer cells (Calu-6). Antibacterial, anti-inflammatory, antioxidant, and cancer-preventive compounds were detected in the MMK01 cells’ methanolic extract following GC–MS analysis. The promising results indicate that *Cyanobium* sp. MMK01 has a great deal of potential for producing C-PC that is on par with strains found in the market, and the tried-and-true two-step purification process proved to work well to achieve an ultra-highly purified form of C-PC.

## Graphical abstract

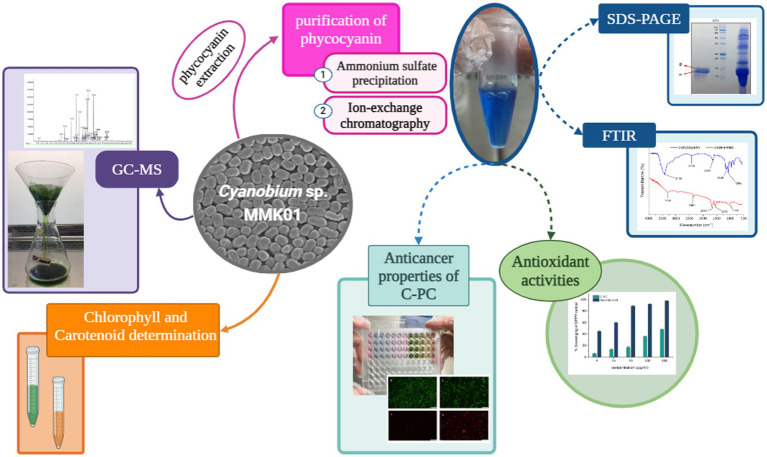

## 1 Introduction

Cyanobacteria are a distinct class of living forms that are the only prokaryotic organisms and the first photosynthetic bacteria that can perform oxygenic photosynthesis and live in a variety of settings (Pagels et al., 2023). These prokaryotic microorganisms have garnered considerable interest from research organizations globally owing to their exceptional capability for carbon fixation and ability to synthesize vast amounts of proteins, lipids, carbohydrates, acids, alcohols, and aliphatic hydrocarbons. The bioactive compounds extracted from them have significant commercial potential due to their potential benefits to human health (Armstrong et al., 2019; Deviram et al., 2020; Aoki et al., 2021). Phycobiliproteins (PBPs) are light-harvesting pigment complexes that are among the many pigments found in these microorganisms (Kaur et al., 2019). According to their optical absorption characteristics, PBPs are categorized into three groups: phycoerythrin (PE), which is red in color with a maximum absorption wavelength (λmax) of 540–570 nm, phycocyanin (C-PC), with a blue hue and λmax of 610–620 nm, and allophycocyanin (APC) with blue-green color and λmax 650–655 nm (Sonani et al., 2014). The water-soluble protein complex C-PC is located on the surface of thylakoids and is involved in the uptake of light energy by cyanobacteria and eukaryotic algae (Puzorjov et al., 2022). Phycocyanins extracted from cyanobacteria (C-phycocyanin) consist of two polypeptide chains: *α* and *β*. The α- and β-polypeptide chains have respective molecular masses of 10–19 and 14–21 kDa. When these subunits are combined, they can form a trimer (αβ3) with a mass of around 120 kDa or a hexamer (αβ6) with a mass of approximately 240 kilodaltons (Kaur et al., 2019; Wang et al., 2023).

C-PC has wide applications in the food, cosmetic, and pharmaceutical industries as an antioxidant, anti-cancer, anti-aging, anti-proliferative, anti-inflammatory, and neuroprotective (Avci and Haznedaroglu, 2022). It is predicted that the lucrative and quickly expanding phycocyanin C-PC market will have grown to USD 245.5 million by 2027. The price per kilogram of this pigment varies depending on its purity, from 360 to 72,460 US dollars (Ashaolu et al., 2021; Hernández Martínez et al., 2023). Many techniques, including chemical treatment (organic and inorganic acids), physical treatment (freeze–thaw, ultrasound, homogenization, and pulsed electric field), enzymatic treatment (lysozyme), and a combination of these techniques, are used to extract C-PC from cyanobacteria (Alotaiby et al., 2024). The extraction efficiency is also influenced by many other variables, including extraction time, solvent-to-biomass ratio, and type of extraction method. The freeze–thaw approach is often suggested as the simplest and most efficient means to extract C-PC from marine or freshwater cyanobacteria (Sivasankari and Ravindran, 2014; Chittapun et al., 2020). It is often necessary to combine more than two purification procedures to extract C-PC from the protein mixture and obtain high-purity C-PC (particularly reagent-grade C-PC with a purity index >4). Typically, ammonium sulfate precipitation and ultrafiltration are used in initial purification, followed by ion exchange chromatography, hydroxyapatite column chromatography, and other column chromatography for repurification. In addition to drastically decreasing the performance of C-PC, the time-consuming processes required to purify it increase production costs. Therefore, the primary objective of current research for C-PC purification is to develop more affordable and efficient purification approaches (Amarante et al., 2020; Scorza et al., 2021; Shi et al., 2024). *Arthrospira platensis* (or *Spirulina platensis*) is the primary source of the most widely used C-PC pigment; nevertheless, there are certain drawbacks to this approach, such as the high cost of the growth medium and the need for multiple extraction and purification steps (Rizzo et al., 2015; Uppin and Dharmesh, 2022). Above all, selecting a potential cyanobacterial strain is a crucial step in producing C-PC effectively. For effective production and utilization, the organism’s nature, growth properties, scaling-up capacity, and, above all, the production yield of C-PC are all crucial factors. Thus, the quest for cyanobacteria that can produce C-PC will continue to be a top focus in biotechnological research (Mogany et al., 2018; Khazi et al., 2021). The primary goals of the current investigation are to extract and purify C-PC pigment from four distinct cyanobacterial strains isolated from the Tehran waterfall and to assess the strains’ C-PC content and purity. Despite numerous reports concerning potential photoautotrophic C-PC producers, little is known about the cyanobacterium *Cyanobium* and strains of this genus for effective C-PC pigment production. In the current study, a potential cyanobacterial strain *Cyanobium* sp. MMK01 was used to obtain a highly purified form of C-PC using only a simple and affordable purification procedure of ammonium sulfate precipitation. Studies were also carried out on the antioxidant properties and effects of C-PC extracted from *Cyanobium* sp. MMK01 on Calu-6 human adenocarcinoma cells. In addition to measuring the amounts of carotenoid and chlorophyll, the methanolic cell extract of *Cyanobium* sp. MMK01 was subjected to GC–MS analysis to identify its valuable chemical compounds.

## 2 Materials and methods

### 2.1 Isolation and molecular identification of cyanobacteria

Samples were taken from the Tehran waterfall in Tehran, Iran (35.7806° = N, 51.2011° = E) to isolate cyanobacteria strains. The samples were cultured in BG11 broth medium at 28°C under continuous aeration with filter sterilized aeration and constant illumination of 70 μEm^−2^ s^−1^ through cold white, fluorescent lamps. After several subcultures on BG11 agar under the same temperature and light conditions, the axenic cultures of each cyanobacterial isolate were attained. Four cyanobacterial isolates were identified using 16S rRNA gene sequencing, and the primers CYA106F (59-CGGACGGGTGAGTAACGCGTGA-39) and CYAN1281R (59-GCAATTACTAGCGATTCCTCC-39) (Valerio et al., 2009). Resulting products were then sequenced by Rajaie Cardiovascular Medical and Research Center, Iran. Sequence similarity searching was done in the NCBI database using the Basic Local Alignment Search Tool (BLAST). The multiple sequence alignment was carried out using the ClustalW program. The phylogenetic tree for four isolated strains was subsequently generated using MEGA11 software via the neighbor-joining method.

#### 2.1.1 Scanning electron microscopy (SEM) analysis

The cyanobacterial cell samples were imaged at high resolution using a scanning electron microscope (SEM) (Hitachi SU-70). Samples were fixed for two hours at 4°C in 0.1 M sodium cacodylate buffer (pH 7.4) containing 3% glutaraldehyde. The cyanobacterial cells were rinsed three times with phosphate buffer before being dehydrated using a graded series of ethanol (10%, 30%, 50%, 70%, and 100%). To improve conductivity, samples were sputter-coated with a thin gold layer (~10 nm) and placed immediately in the chamber following preparation.

### 2.2 Growth profile of cyanobacterial strains

In order to standardize the test conditions, an equal weight of cyanobacterial biomass was inoculated into 2-liter Erlenmeyer flasks containing 500 mL of BG11 culture medium and incubated at 28°C under constant illumination of 70 μEm^−2^ s^−1^. The optical density at a wavelength of 750 nm was measured over consecutive days to track the growth rate of the desired strains and determine the best time to harvest the cyanobacterial cells. The dry cell weight (DCW) was determined in line with the optical density after measuring the OD of homogenized cyanobacteria in the early, middle, late, and stationary growth phases. In each growth stage, the cell-free supernatant (CFS) was obtained through centrifugation (6,000 × g at 4°C for 15 min) of liquid culture medium. Then, the collected pellet was washed with distilled water twice, followed by freeze-drying. Finally, the dry weight of the biomass of cyanobacterial strains in each of the desired phases was measured in terms of mg/L.

### 2.3 Purity and quantity of C-PC

The purity and concentration of C-PC in different stages of extraction and purification, respectively, were obtained by dividing OD620 by OD280 and using the following equation (Bennett and Bogorad, 1973).


$$\mathrm{Concentration}\;\mathrm{of}\;C-PC\;\left(\mathrm{mg}/\mathrm{mL}\right)=\frac{A620-\left(0.474\times A650\right)}{5.34}$$

The production yield of C-PC was calculated according to the following equation (Silveira et al., 2007).


$$\mathrm{Yield}\left(\mathrm{mg}/g\right)=\frac{\left(C-PC\right)V}{DB}$$

Where C-PC represents C-PC content (mg mL^−1^), V solvent volume (mL) and DB dried biomass (g).

### 2.4 C-PC synthesis with cell growth

To investigate the ideal time to harvest cyanobacteria cells for the efficient production of C-PC pigment, equal to 33 mg of dried biomass was removed in each of the desired growth phases. In the next step, one milliliter of water was added to each sample and subjected to freeze–thaw cycles, followed by centrifugation (9,000 × g for 15 min, 4°C). The absorbance of the resultant supernatant in each growth phase was then read at a wavelength of 200–800 nm. Finally, the most appropriate growth phase was determined for biomass harvesting to produce optimal C-PC. In addition, the strain with the highest concentration and purity of C-PC was selected for further studies.

### 2.5 Extraction of C-PC from the selected strain, *Cyanobium* sp. MMK01

#### 2.5.1 Freeze–thaw method

A biomass-to-solvent (distilled water) ratio of 1:30 was employed to extract C-PC using the freeze–thaw procedure. This was accomplished by dissolving 1 gram of dry biomass in thirty milliliters of distilled water, freezing it for 4 h at −70°C, and then thawing it for 4 h at −4°C. Subsequently, the resulting solution was centrifuged at 9,000 x g for 15 min at 4°C. Lastly, the purity of C-PC was determined by measuring the absorbance of the generated supernatant. Following three cycles of freezing and thawing, the supernatant was collected and examined for C-PC content and purity.

### 2.6 Purification of C-PC from *Cyanobium* sp. MMK01

#### 2.6.1 Ammonium sulfate precipitation

Ammonium sulfate salt with a specific concentration was gradually added to the solution in each step. For this purpose, 30% ammonium sulfate (Merck, Germany) was added to 10 mL of crude extract for protein precipitation, followed by centrifugation at 9,000 x g for 15 min at 4°C, then the supernatant was separated. In the second step, ammonium sulfate powder was added to the separated supernatant until the percentage of salt in the solution reached 40%, and the absorbance of the supernatant was measured in each step at a wavelength of 200–700 nm by a spectrophotometer (Bioanalytic, Jena, Germany). By calculating the purity of C-PC, the best percentage of ammonium sulfate was selected for further purification.

#### 2.6.2 Anion exchange chromatography

The precipitate solution (previous step) was dialyzed against phosphate buffer (10 mM, pH 7.5) at 4°C overnight. It was then loaded onto a 1.2 × 10 cm column packed with Q-Sepharose XL anion exchange resin (GE Healthcare, Germany). The column was pre-equilibrated with phosphate buffer. According to the program shown in Table 1, an elution flow rate of 3 mL/min was carried out with a multiple-slope gradient of 0 to 0.5 M NaCl solution in phosphate buffer (10 mM, pH 7.5). 1 mL fractions were collected, and their absorption was measured in the wavelength range of 200–700 nm. The resulting absorption spectra were used to determine the purity and concentration of C-PC (eluting fluid captured over fraction collection). After pooling and dialysis against distilled water, fractions with a purity index higher than 4 were freeze-dried and used for bioassays and subsequent analytical experiments.

**Table 1:** Multi-gradient washing program for purification of C-PC by ion exchange chromatography.

Time (min)	Buffer A (%)	Buffer B (%)
30	100	0
50	80	20
70	80	20
220	70	30
270	0	100

### 2.7 Determination of C-PC pigment characteristics

#### 2.7.1 C-PC size determination

Sodium dodecyl-sulfate polyacrylamide gel electrophoresis (SDS-PAGE) was carried out under denaturing conditions to ascertain the molecular mass of each C-PC subunit and assess the effectiveness of the purification procedure. SDS-PAGE gel (15%) electrophoresis was performed at 80 V for 30 min and then at 120 V to complete the run. The Coomassie blue R250 stain was used to visualize the proteins.

#### 2.7.2 FTIR analysis

The compositional features of C-PC were analyzed using FTIR spectroscopy by Perkin-Elmer Spectrometer (FTIR GX 2000). For this purpose, before conducting an FTIR analysis, the dried C-PC powder (both before and after purification) was thoroughly combined with potassium bromide (KBr) (Sigma-Aldrich, USA) in a ratio of 1:50 (w/w). These samples were compressed into pellets using a 10-ton pressure load using a hydraulic pellet press to obtain transparent pellet samples that allow the IR radiation to pass through them. The spectra were taken in the range of 400–4,000 cm^−1^ with 8 cm^−1^ resolution.

#### 2.7.3 Analysis of C-PC’s anticancer properties

Calu-6 human lung cancer cell line was cultured in Dulbecco’s Modified Eagle Medium (DMEM; Bioidea, BI-1001, Iran) culture medium containing 10% fetal bovine serum (FBS; BI-1201, Bioidea, Iran) to assess the anti-proliferative characteristics of C-PC. The cells were seeded with a cell density of 10^4^ cells per well on a 96-well microplate. The consecutive wells (in a total volume of 100 mL culture media) were established with C-PC concentrations of 5, 10, 15, 20, 30, and 40 μg/mL. At 24, 48, and 72 h, the cytotoxic effect of various C-PC concentrations was tested independently. Calu-6 cells were kept in an atmosphere of 5% CO_2_ and 95% humidity at 37°C. Following C-PC treatment, 10 μL of 0.5% MTT solution in PBS were added to each well, and they were then incubated for an additional 4 h at 37°C. Then the supernatant was removed, and the cells were incubated in 100 μL dimethyl sulfoxide (DMSO; Merck, Germany) for 30 min. The viability of the treated cells was assessed by measuring the absorbance at 570 nm using a microplate reader (Lab-Systems Multiskan, Roden, Netherlands). The half-maximal inhibitory concentration (IC_50_) was calculated at different times. The human fibroblast cells and Calu-6 were then similarly treated for 24, 48, and 72 h with the IC50 concentration of C-PC to examine the effect of C-PC on normal and cancer cells. The experiments were conducted three times.

#### 2.7.4 Assessment of cell membrane integrity and viability using FDA/PI staining method

The effect of C-PC on the membrane integrity of cancer cells stained with FDA/PI was evaluated by fluorescence microscopy (Nikon Eclipse-Ti, Japan). Cells were seeded at the same density, as mentioned above, for 24 h, while C-PC was applied at IC50. After the incubation period, 100 μL of FDA solution was added to the wells, and imaging was done under a fluorescence microscope at an emission wavelength of 510 nm and an excitation wavelength of 493 nm after 10 min. After that, 30 μL of PI solution was added to each well, and images were taken in the dark. The FDA staining solution was prepared by combining 10 mL of phosphate-buffered saline (PBS) with a 0.5% acetone solution and 40 μL of FDA. PI was dissolved in PBS to make a 0.002% stain solution.

### 2.8 Antioxidant activity of C-PC by DPPH radical scavenging approach

The antioxidant activity of C-PC was measured following the change in color of the 2,2-diphenyl-1-picrylhydrazyl (DPPH) solution in methanol (Merck,Germany). For this purpose, 3 mL of C-PC in different concentrations of 25, 50, 100, and 200 μg/mL and ascorbic acid, as a standard, were mixed with 1 mL of 0.01 mM DPPH solution. The reaction was allowed to proceed for 30 min without any light, and then the absorbance at a wavelength of 517 nm was determined. The percentage of antioxidant activity was calculated using the following equation (Renugadevi et al., 2018).


$$\mathrm{Scavenging DPPH}=\left[\frac{\left(A0-A1\right)}{A0}\right]\times 100.$$

where A0 is DPPH absorbance without pigment sample and A1 is absorbance containing pigment sample with DPPH.

### 2.9 Preparation of methanol extract of *Cyanobium* sp. MMK01 cells for GC–MS analysis

*Cyanobium* sp. MMK01 biomass was dried in a hot air oven and 2 g of powdered biomass was soaked in 95% methanol for 12 h. Then, the extract was filtered through Whatman 41 filter paper along with 0.2 g of sodium sulfate to remove sediments and water traces. Before filtering, the filter paper was moistened with 95% ethanol for 12 h. Then, by introducing nitrogen gas bubbles into the solution, the filtration was concentrated. Finally, 2 mL of this solution was used for GC–MS analysis (Ebadi et al., 2019).

#### 2.9.1 GC–MS analysis

The gas chromatography–mass spectrometry (GC–MS) profiling of active fraction was performed using Agilent 6,890 GC coupled with an MS detector (Agilent Technologies, USA). High-purity helium (99.99%) was utilized as the carrier gas, with an injection volume of 1 μL (split ratio of 1:5) and an injector temperature of 280°C. The gas was used at a constant flow rate of 1 mL/min. The oven temperature was set to climb at a rate of 5°C per minute from 80°C (isothermal for 2 min) to 280°C (isothermal for 5 min). Mass spectra at 70 eV and a scan mass range of 40–500 m/z were employed. Data acquisition was performed by comparing with mass spectra as a possible match in the NIST/Wiley library search.

### 2.10 Measurement of carotenoid and chlorophyll

After being weighed, 15 mg of wet biomass was transferred into a 2 mL vial. The vial was then submerged in liquid nitrogen for 15 min. Subsequently, the container was filled with 1 mL of methanol, vortexed, and centrifuged at 10,000 x g for 10 min. Ultimately, using the following formulas, the amount of carotenoid and chlorophyll synthesis was determined by measuring the OD of the supernatant at wavelengths 461 and 664 nm (Chamovitz et al., 1993).


$$\begin{array}{l} \mathrm{Carotenoid concentration}\;\left(\mathrm{\mu g}/\mathrm{mL}\right)=\left[O.D.461–\left(0.046\times O.D.664\right)\right]\\ \times \mathrm{4Chlorophyll concentration}\;\left(\mathrm{\mu g}/\mathrm{mL}\right)=O.D.664\times 11.92 \end{array}$$

## 3 Results

### 3.1 Phylogenetic analysis and characteristics of isolated strains

**Figure 1:**
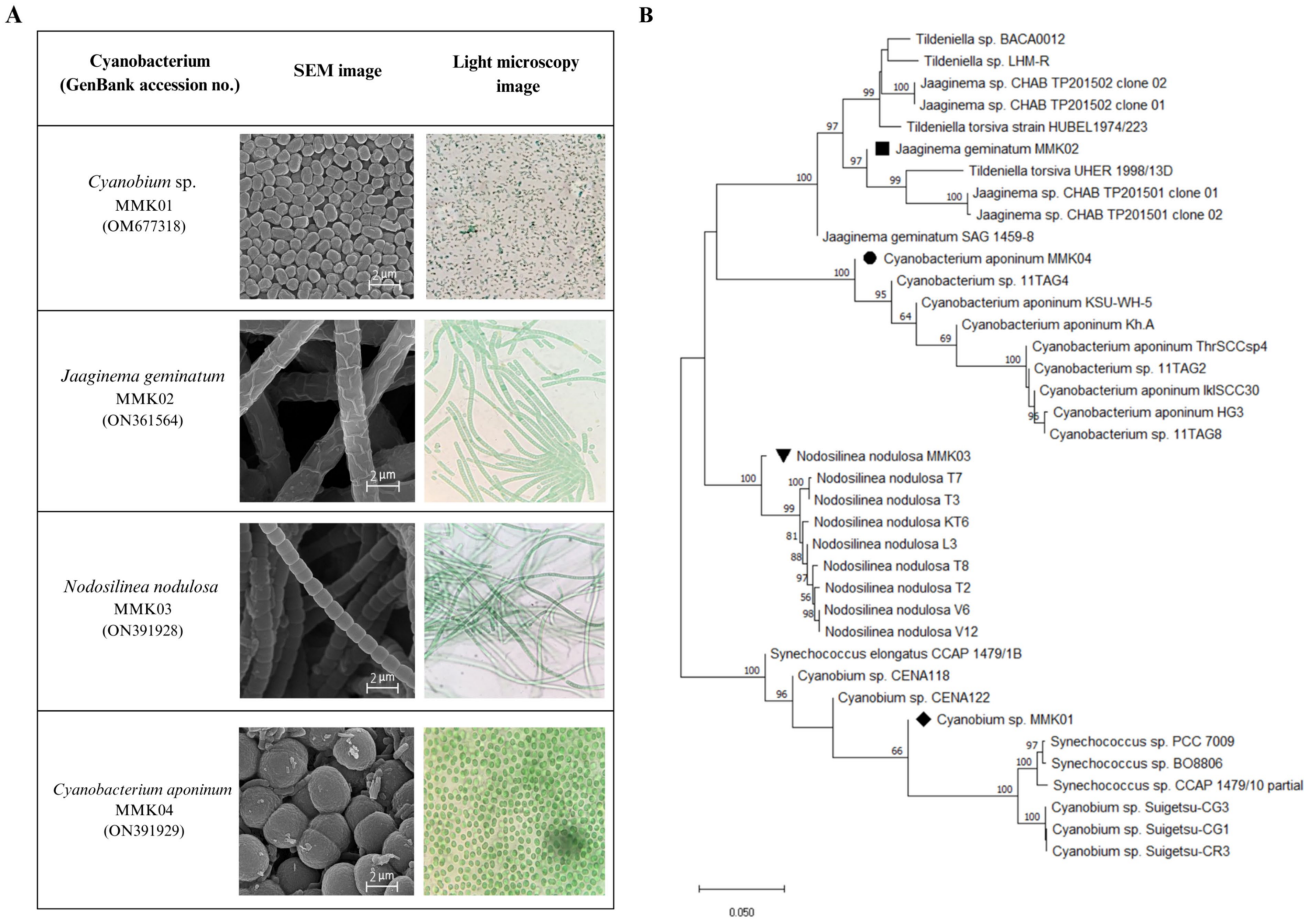
(A) Light and scanning electron microscope (SEM) images of four cyanobacterial strains isolated from Tehran waterfall. (B) Phylogenetic tree based on 16S rRNA gene sequencing of four strains of cyanobacteria.

Four different cyanobacteria strains were isolated from the collected samples based on the difference in initial appearance characteristics, such as colony shape and microscopic images. The light and electron microscope images of these strains have been shown in (Figure 1A). The initial morphological analysis of the cyanobacterial isolates using an optical microscope and scanning electron microscope (SEM) showed, that of four bacterial isolates, two have filamentous-like structures with different diameters, and the other two displayed a spherical shape with varied dimensions. Furthermore, the molecular identification was carried out by partial 16S rRNA gene sequences, and the obtained sequence was compared with the existing sequences in the NCBI database by the BLAST. The BLAST analysis of the corresponding sequences from the Furthermore, the molecular identification was carried out by partial 16S rRNA gene sequences, and the obtained sequence was compared with the existing sequences in the NCBI database by the BLAST. The BLAST analysis of the corresponding sequences from the four cyanobacterial isolates revealed that they had, respectively, 99, 99, 98, and 96% identity with the reference sequences of the *Jaaginema geminatum, Cyanobium* sp.*, Nodosilinea nodulosa,* and *Cyanobacterium aponinum*. The phylogenetic tree was constructed by neighbor-adjusting sequence alignment with 1,000 bootstrap replicates and the p-distance substitution model (Figure 1B). The 16S rRNA gene sequence of the cyanobacterial isolates was submitted to NCBI and registered with the names and accession numbers of *Cyanobium* sp. MMK01 (0 M677318), *Jagginema geminantum* MMK02 (ON361564), *Nodosilinea nodulosa* MMK03 (ON391928), and *Cyanobacterium aponinum* MMK04 (ON391929).

### 3.2 Cyanobacterial cell growth and biomass

**Figure 2:**
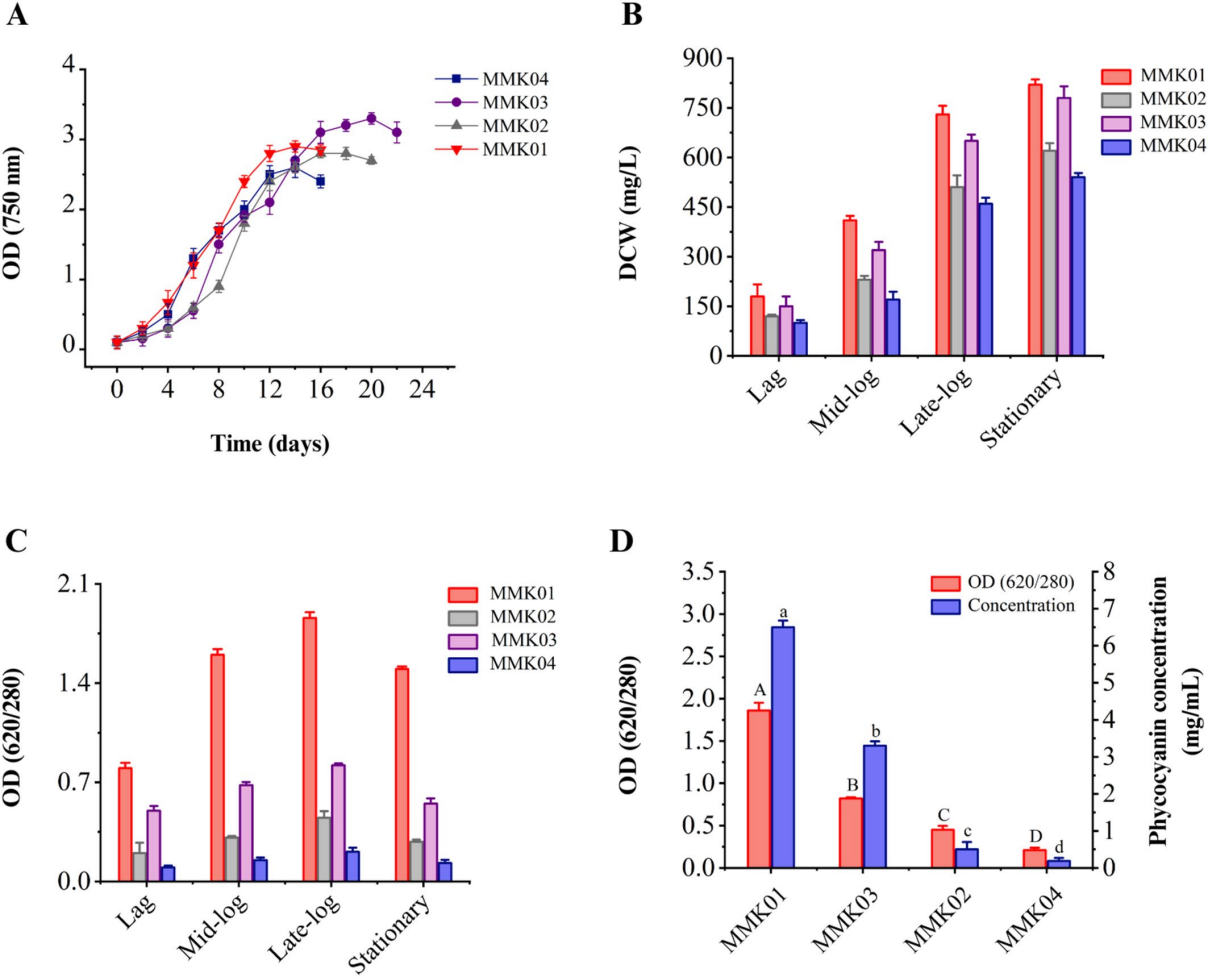
(A) The growth profile of four cyanobacteria isolates was determined by measuring OD 750 nm during consecutive days. (B) The dry cell weight content (mg/L) at every stage of the growth phase. Values are shown as mean ± standard deviation with three replicates. (C) Analysis of the purity of C-PC in each phase of cell growth, including lag, mid-log, late-log, and stationary phase. (D) Purity and concentration of C-PC of four cyanobacterial strains at the end of the growth phase. Values are shown as mean ± standard deviation with three replicates. Different small letters and capital letters above the bars represent statistically significant differences in concentration and purity of C-PC, respectively (one-way ANOVA; Tukey test; *p* < 0.05).

Analysis of the growth profiles of four cyanobacteria strains revealed that biomass and cell growth increased with increasing incubation time and peaked at the end of the logarithmic phase. Each cyanobacterial strain had a different growth profile and reached the end of the logarithmic phase on a specific day of its incubation (Figure 2A). The changes of dry weight biomass in each phase of the growth curve, including the lag phase, mid-exponential growth phase, late exponential phase, and stationary phase, are shown in Figure 2B. The results show an increase in the dry cell weight in parallel with the cell growth.

### 3.3 Cyanobacteria growth and investigating the purity of C-PC

During the bacterial growth phase (from the lag phase to the stationary phase), the variations in C-PC purity to biomass dry weight were investigated. As shown, in parallel with cell growth and biomass increase, the production and purity of C-PC have also increased, and the highest level of C-PC purity was achieved at the end of the growth phase (Figure 2C). The purity rate of C-PC was decreased when the cell reached the stationary phase of growth.

### 3.4 Purity and concentration of C-PC and selecting the best-producing strain

The purity and concentration of C-PC are two critical parameters in selecting the best- producing strain. Therefore, a comprehensive study was conducted to assess the purity and concentration of C-PC in the crude extract of four cyanobacterial strains using a single freeze-thaw extraction method, similar to the procedure established for phycocyanin extraction. The crude extract of *Cyanobium* sp. MMK01 reached its peak growth in fewer days compared to the other three strains, demonstrating higher C-PC purity and concentration in the crude extract. As a result, this strain was selected for further investigation (Figure 2D).

### 3.5 Optimal freeze–thaw cycle for the extraction of C-PC from *Cyanobium* sp. MMK01

**Figure 3:**
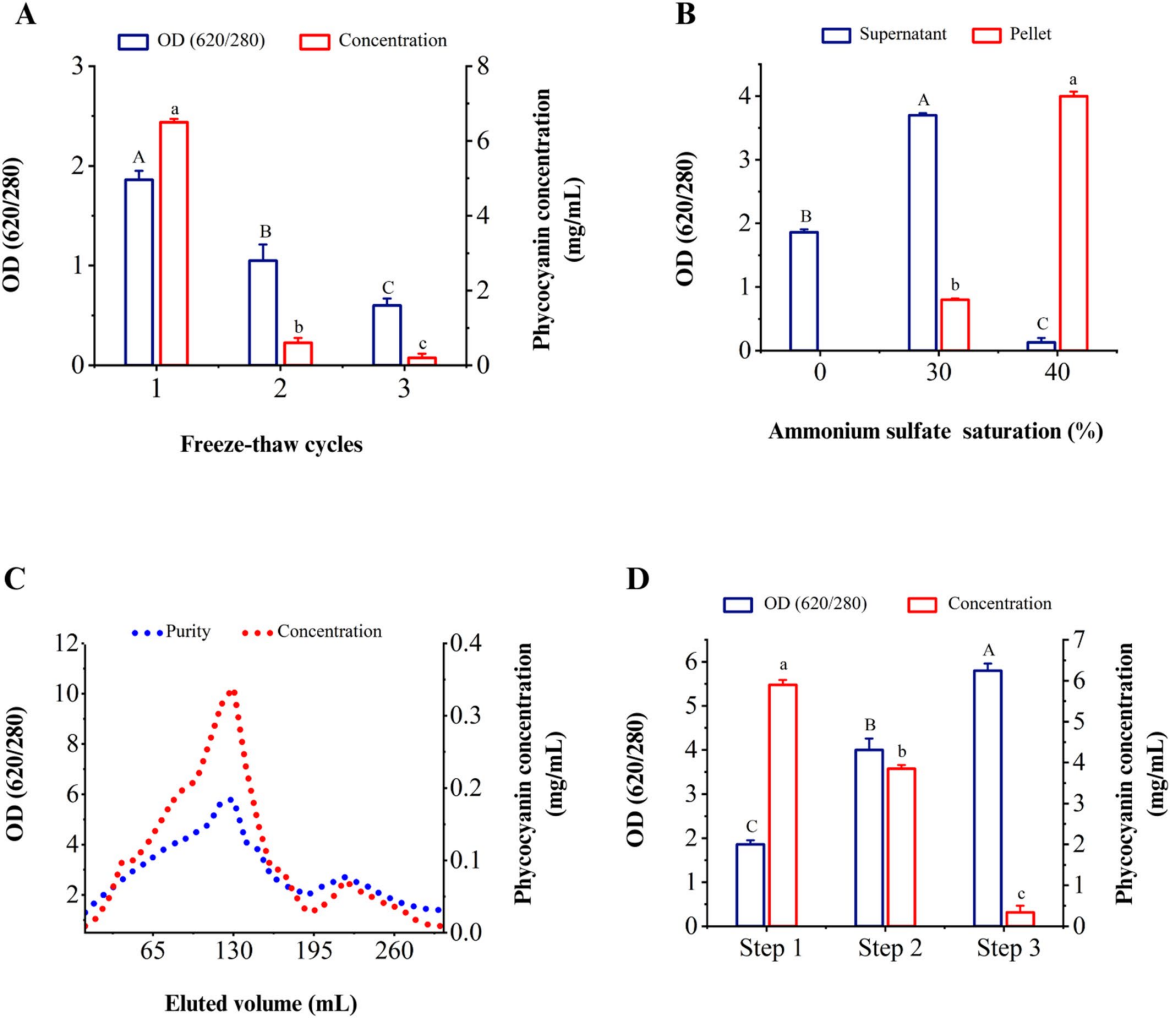
Purity and concentration of C-PC in different stages of extraction and purification. (A) Different cycles of freezing and thawing to extract C-PC, (B) the effect of ammonium sulfate saturation on the purity of C-PC in the sediment and supernatant, (C) the purity and concentration of C-PC in the washed fractions through ion exchange chromatography. (D) successive stages of extraction and purification of C-PC from *Cyanobium* sp. MMK01. Step 1. C-PC extraction by freezing and thawing step 2. Purification by ammonium sulfate precipitation step 3. Purification by ion chromatography method. Values are shown as mean ± standard deviation. *n* = 3; Different small letters and capital letters above the bars represent statistically significant differences in concentration and purity of C-PC, respectively (one-way ANOVA; Tukey test; *p* < 0.05).

The optimal method of C-PC extraction is an essential parameter to produce this pigment from cyanobacteria. Therefore, the purity and concentration of C-PC were calculated in different freeze–thaw cycles of dissolved-water biomass. Based on the results, the highest values of purity and concentration of C-PC were obtained in the first freeze–thaw cycle. The concentration and purity of C-PC considerably decreased compared to the initial cycle of freezing and thawing subsequently (Figure 3A).

### 3.6 Purification of C-PC from *Cyanobium* sp. MMK01

#### 3.6.1 Ammonium sulfate precipitation

As the concentration of ammonium sulfate (AS) increased from 0 to 30%, the purity index of C-PC in the solution increased based on the results of various percentages of AS. The maximum purity of C-PC was obtained when the concentration of AS reached 40%. As illustrated in Figure 3B, the purity of C-PC increased from 1.86 to 4.04 following the first purification stage.

#### 3.6.2 Purification by ion-exchange chromatography

From fraction 80 to fraction 140, the purity of C-PC was more than 4, reaching its peak at nearly 5.82 in fraction 130. From fraction 150 to fraction 190, the purity of C-PC decreased until, at last, it increased again in fraction 220. The second peak, eluted after the C-PC, was related to allophycocyanin with a specific absorbance at 650 nm, increasing the absorbance ratio of 280/620 (Figure 3C). Figure 3D shows that the freeze–thaw method for extracting C-PC gives the highest concentration, while the use of the ion exchange chromatography technique leads to the highest purity of C-PC. However, following the extraction of C-PC by freeze–thaw, the purification of C-PC using the ammonium sulfate precipitation method yielded a comparatively high concentration and purity of 3.85 mg/mL and 4.04 form of C-PC, respectively.

### 3.7 Determination of molecular mass

**Figure 4:**
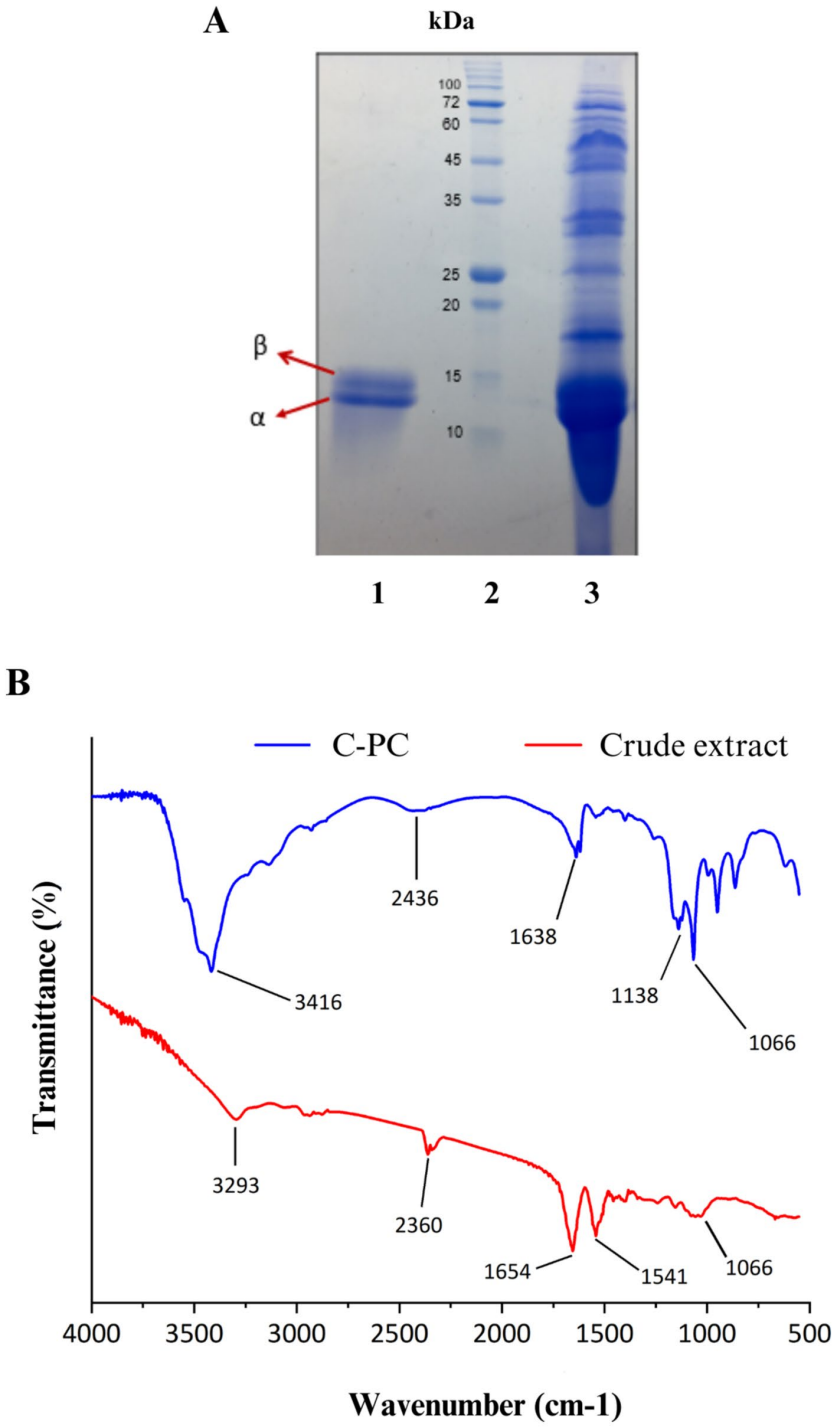
(A) SDS-PAGE analysis of C-PC purified from *Cyanobium* sp. MMK01. Lane1: *α* and *β* subunits of C-PC stained with Coomassie brilliant blue with a molecular weight of about 13 kDa and 15 kDa, respectively, Lane 2: protein marker, Lane 3: crude extract of *Cyanobium* sp. MMK01. (B) FT-IR analysis of crude extract and purified C-PC from *Cyanobium* sp. MMK01.

In order to check the accuracy of purification and identification of C-PC pigment subunits, the purified pigment from *Cyanobium* sp. MMK01 was loaded on SDS-PAGE gel. Two C-PC subunits (alpha and beta) were separated from each other on SDS-PAGE gel with a molecular mass of approximately 13 kDa and 15 kDa, respectively. Comparison of C-PC before and after purification (on the SDS-PAGE gel) was consistent with the spectrophotometric results, indicating the high purity of the C-PC product after purification (Figure 4A).

### 3.8 Fourier transform infrared spectroscopy (FTIR) analysis

The FTIR analysis was conducted to identify the functional groups present in C- phycocyanin (C-PC) pigment, purified from the crude extract of the cyanobacterium *Cyanobium* sp. MMK01, within the spectral range of 600 -4000  cm^−1^ (Figure 4B). In the crude phycocyanin, a prominent peak at 3293  cm^−1^ was detected, which corresponds to the stretching vibrations of OH and NH groups, likely indicating the presence of water and hydrogen bonds. After purification, this band shifted to 3416   cm^−1^, suggesting a reduction in water content and The removal of impurities associated with hydrophilic molecules. The Amide I band, representing the stretching vibrations of the C=O bond in the peptide backbone, was observed at 165  cm^−1^ in crude phycocyanin. This band shifted to 1638   cm^−1^ following purification, indicating alterations in the secondary structure of the protein. This detailed FTIR analysis demonstrates the structural and chemical changes in C-PC after purification, confirming the enhancement in protein purity and structural order.

### 3.9 Anticancer properties of C-PC

#### 3.9.1 Inhibitory effect of C-PC on human lung carcinoma cells (Calu-6) proliferation

**Figure 5:**
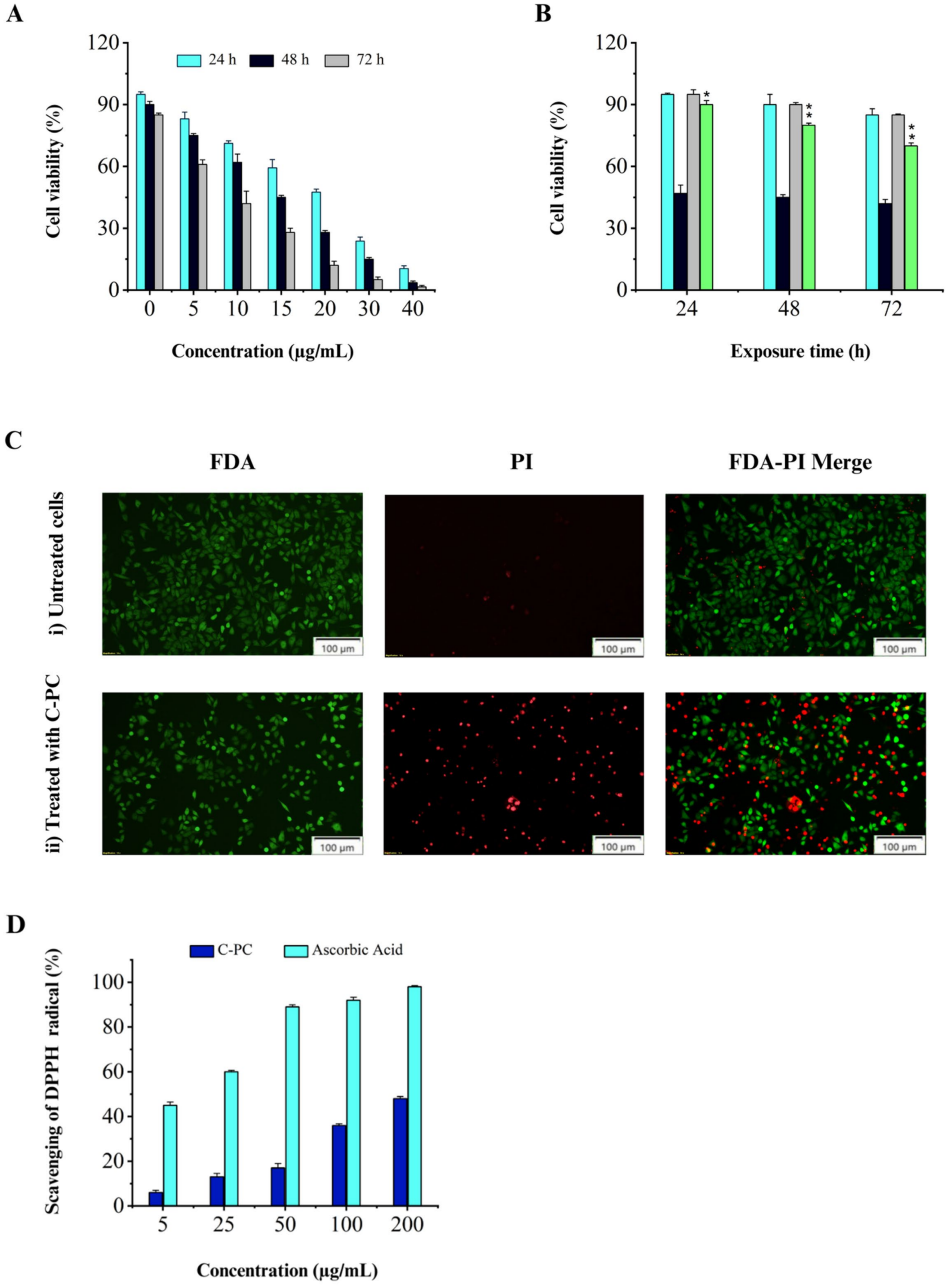
Evaluation of cytotoxicity and antioxidant activity of purified C-PC. (A) MTT assay results obtained from the treatment of Calu-6 cells with different concentrations of C-PC for 24, 48, and 72 h, (B) Comparing the MTT assay results of human fibroblasts and Calu-6 cells following 24, 48, and 72 h of treatment with C-PC at the corresponding IC50 concentration. Values are shown as mean ± standard deviation. Asterisks above each bar represent differences from full-length control (one-way ANOVA; Holm-Sidak test; *p < 0.05; **p < 0.01). (C) Fluorescence microscopy images of FDA/PI-stained Calu-6 cells, (i) cells not treated with C-PC, (ii) cells treated with C-PC. Scale bar = 100 μm. (D) Antioxidant activity of pure C-PC extracted from *Cyanobium* sp. MMK01.

The results showed that C-PC affected the cells in a time- and dose-dependent manner. A higher inhibitory effect on cell proliferation was observed with increasing C-PC concentration or period of time. The IC50 values of the C-PC treatment are shown in (Figure 5A) at concentrations of 10, 15, and 20 μg/mL for 24, 48, and 72 h, respectively. However, after being exposed to C-PC for 24, 48, and 72 h, human fibroblast cells did not exhibit any adverse effects at IC50 concentration (Figure 5B).

#### 3.9.2 Analysis of cell membrane integrity and function by FDA and PI staining

Living cells are observed using FDA staining in green fluorescence, while dead cells are visible using PI staining in red fluorescence. According to Figure 5C, the IC50 concentration of C-PC caused a considerable decrease in the live/dead cell ratio in Calu-6 cells, and almost 50% of the cells turned red. These results suggest that C-PC, which was isolated from *Cyanobium* sp. MMK01 is detrimental to lung cancer cells.

### 3.10 Antioxidant activity of C-PC by DPPH radical scavenging approach

The DPPH free radical scavenging assay is based on the reduction of DPPH to 2,2-diphenyl-1-picrylhydrazine (DPPH-H) (yellow color), indicating the availability of a hydrogen donor. Ascorbic acid was used as a positive control. The results showed that C-PC with a concentration of 200 μg mL^−1^ can inhibit DPPH radicals up to 48% (Figure 5D).

### 3.11 Gas chromatography–mass spectrometry (GC–MS) analysis

GC–MS analysis of the methanolic extract of *Cyanobium* sp. MMK01 revealed the presence of 39 distinct bioactive compounds (see Supplementary material). According to GC–MS analysis, n-hexadecanoic acid (20.86%), 11(Z)-hexadecenoic acid (19.41%), tetradecanoic acid (10.79%), phytol (6.86%), and heptadecane (6.31%) were the main constituents in the methanol extract of *Cyanobium* sp. MMK01. These substances possess anti-inflammatory, antimicrobial, antioxidant, and anti-cancer properties (Table 2).

**Table 2:** List of compounds identified by GC–MS analysis in the methanolic extract of *Cyanobium* sp. MMK01.

RT	Name of the compound	Peak area%	Molecular formula	Class	Activity
21.57	Heptadecane	6.31	C_17_H_36_	Aliphatic	Antioxidant
22.94	Tetradecanoic acid (myristic acid)	10.79	C_14_H_28_O_2_	Acid	Antioxidant, antimicrobial
26.49	Hexadecenoic acid, Z-11-	19.41	C_16_H_30_O_2_	Acid	Antimicrobial
26.92	n-Hexadecanoic acid (Palmitic acid)	20.86	C_16_H_32_O_2_	Acid	Antioxidants, hypocholesterolemic, and nematicide,
29.41	Phytol	6.86	C_20_H_40_O	Alcohol	Antimicrobial, anticancer and anti-inflammatory

### 3.12 Chlorophyll and carotenoid determination

While the purification and analysis of C-PC from *Cyanobium* sp. MMK01 was the main objective of the present study, the amounts of other pigments, such as carotenoids and chlorophylls, were also evaluated. After being extracted, the amount of carotenoid and chlorophyll in *Cyanobium* sp. MMK01 was determined to be 10.75 μg mL^−1^ and 35.71 μgmL^−1^, respectively. We came to the conclusion that the MMK01 strain had a significant pigment concentration.

## 4 Discussion

C-PC has wide applications in food, health, and pharmaceutical industries due to its anticancer, antibacterial, antiviral, and anti-inflammatory properties (Khazi et al., 2021; Fernandes et al., 2023). It is worth noting that the extraction and purification of C-PC from cyanobacteria lacks an established methodology. C-PC in its purest form has been obtained in recent studies using various methods. These multi-step purification processes are costly and time-consuming (Yu et al., 2017; Sintra et al., 2021). Thus, there is a substantial economic advantage in employing simple and affordable techniques to achieve high concentration and purity of C-PC. In the course of our research, we were able to identify and isolate a novel and productive strain of cyanobacteria, developing a low-cost, simple-to-operate process for producing highly purified C-PC. *Cyanobium* sp. MMK01 (OM677318), which exhibited the highest concentration and purity of C-PC, was ultimately determined to be the best strain following a thorough investigation of the four isolated cyanobacterial strains. The number of freeze/thaw cycles required for extracting C-PC varies depending on the type of cyanobacteria. To prevent cyanobacterial cells and their protein content from being damaged during the extraction process, the cyanobacterial strain’s structure, in particular, needs to be taken into account. It has been found that the C-PC concentration and purity are significantly impacted by the number of freeze–thaw cycles applied to the extraction solution. For instance, extracting C-PC from *Arthrospira platensis* required one to three freeze–thaw cycles, however, extracting C-PC from the *Nostoc commune* strain TUBT05 required more than three freeze–thaw cycles to achieve the desired concentration and purity (Minkova et al., 2007; Tavanandi et al., 2018; Chittapun et al., 2020; Roy and Pabbi, 2022). Increased freeze–thaw cycles have the potential to damage proteins and cells, generate ice crystals, and lower the purity of C-PC while raising the level of organic contaminants in the crude extract (Chittapun et al., 2020; Avci and Haznedaroglu, 2022). While requiring more time than other standard procedures and solvents, extraction of C-PC pigment using water can be considered an appealing and cost-effective substitute (Aoki et al., 2021). Using water as a solvent and just one freeze–thaw cycle, the highest concentration and purity of C-PC from the *Cyanobium* sp. MMK01 was obtained in this study. The MMK01 strain has superior characteristics to the other strains studied thus far because it requires less time to obtain high concentration and purity of C-PC during the extraction process (Kaur et al., 2019).

Based on the C-PC purity index, this product is graded differently for different uses and purposes. While C-PC with an index of 3.9–4 or above can be utilized in medical biotechnology as a fluorescent label or antioxidant in pharmaceutical products, a purity index of at least 0.7 allows the product to be used as a colorant in food and cosmetics (Eriksen, 2008; Antecka et al., 2022; Hernández-Martínez et al., 2023). A highly pure form of C-PC with a purity index of 5.26 was achieved in a prior study using multiple sequential procedures for the purification of C-PC from *Limnothrix* sp. NS01 (Safaei et al., 2019). Table 3 indicates that the present study is particularly significant compared to earlier studies. Here, we achieved purifying C-PC from *Cyanobium* sp. MMK01 with high concentration and purity while simultaneously developing a less complex and more affordable purification process. From an industrial perspective, where the purification process incurs significant costs, this point is of great importance. In the second step, C-PC from *Cyanobium* sp. MMK01 was purified using the ion chromatography method, yielding a purity of 5.82. This level of C-PC purity has rarely ever been reported in prior research (Mahendran et al., 2022) (Table 3). Apart from achieving an exceptional level of C-PC purity, this study additionally demonstrated that it could generate C-PC at a greater concentration and production efficiency compared to earlier research published so far (Avci and Haznedaroglu, 2022; Hernández–Martínez et al., 2023; Prabha et al., 2023; Sánchez-Laso et al., 2023). The present study is the first of its kind since no prior research has been conducted on the development of an appropriate and ideal method for the extraction and purification of the highly purified form of C-PC (purity index 5.82) with a high concentration from *Cyanobium* sp. Therefore, compared to other potential cyanobacterial strains, like *Spirulina* sp., the *Cyanobium* sp. strain MMK01 may perform better from a commercial standpoint for large-scale C-PC production.

**Table 3:** A comparison of the present study with earlier research regarding the different methods employed to extract and purify C-PC from various cyanobacteria.

Strain	Extraction				Purification method				References
	Method	C-PC concentration (mg mL^−1^)	C-PC purity ratio	Yield (mg C-PC g DCW^−1^)	Method	C-PC concentration (mg mL^−1^)	C-PC purity ratio	Yield (mg C-PC g DCW^−1^)	
*Desertifilum tharense* UAM-C/S02	repeated FT	0.822	1.40	86.46	Two-step ASP+ Dialysis	0.576	3.83	ND	Hernández Martínez et al. (2023)
*Synechocystis* sp.	BB	ND	1.15	182.4	AEC	ND	4.84	16.9	Avci and Haznedaroglu (2022)
*Phormidium* sp.	FT	ND	0.55	48.1	ASP	ND	1.75	33.0	Avci and Haznedaroglu (2022)
*Euryhalinema* sp.	Ultrasonication + FT	ND	0.76	15.02	ASP + Sephadex-G25 + DEAE-Sephadex	ND	4.1	ND	Khazi et al. (2021)
*Leptolyngbya* sp.	MP + three cycles of FT	0.038	0.36	ND	Three-stage ASP + AEC	0.092	3.5	ND	Prabha et al. (2023)
*Synechocystis* sp. PCC 6803	High pressure homogenisation	ND	1.25	75.3	Two-step ASP + heat-treatment	ND	2.9	ND	Puzorjov et al. (2022)
*Spirulina* sp.	The enzyme treatment	0.081	0.93	ND	ASP + Dialysis + DEAE column chromatography	0.545	5.02	ND	Mahendran et al. (2022)
*Arthrospira platensis*	[EMIM][EtSO4] ionic liquid + sonication	ND	0.54	76.6	dialysis + precipitation	ND	3.5	48.9	Sánchez-Laso et al. (2023)
*Spirulina*	3 cycles of FT	ND	0.77	217.18	-	-	-	-	Chentir et al. (2018)
*Spirulina*	4 cycles of FT	ND	0.66	73.73	-	-	-	-	Tavanandi et al. (2018)
*Spirulina*	Soaked in potassium phosphate buffer	ND	0.93	17.2	chitosan + activated charcoal + chromatography on DEAE Sephadex A-25	ND	4.30	ND	Liao et al. (2011)
*Cyanobium* sp. MMK01	1 cycles of FT	6.5	1.86	225	Two-step ASP	3.85	4.04	120	This study
*Cyanobium* sp. MMK01	1 cycles of FT	6.5	1.86	225	Two-step ASP +IEC	0.34	5.82	6.8	This study

FT, Freeze/thaw Cycles; BB, Bead-beating; MP, mortar and pestle; ASP, ammonium sulfate precipitation; AEC, Anion-exchange chromatography; IEC, ion exchange chromatography; DEAE, diethyl aminoethyl; HPLC, high-performance liquid chromatography. ND: Not determined. *Phycocyanin purity index: A615-620/A280 > 0.7—food grade; >1.5—cosmetic grade, >3.9—reagent grade, >4.0—analytical grade.

In a previous study by Gantar et al. (2012), the molecular mass of C-PC from *Limnothrix* sp. strain 37–2-1 was determined to be roughly 11 kDa and 13 kDa for the *α* subunit and *β* subunit, respectively (Gantar et al., 2012). In another study, pure C-PC from *Plectonema* sp. was subjected to SDS-PAGE analysis and revealed two bands with molecular masses of 17 kDa and 19 kDa, respectively, corresponding to the α and β subunits (Husain et al., 2021). The molecular masses of the α and β subunits identified in this study using SDS-PAGE analysis were approximately 13 kDa and 15 kDa, respectively. This indicates that molecular masses of C-PC generated by various cyanobacterial strains can vary greatly (Kaur et al., 2019).

Because of the adverse effects of chemical anticancer medications, the use of natural products to prevent carcinogenesis has grown to become increasingly significant in cancer research in recent years. Bioactive compounds derived from cyanobacteria have received significant attention from the scientific community due to their strong anticancer properties. C-PC pigment has been demonstrated in numerous studies to have advantageous biological characteristics, such as anti-inflammatory and anti-cancer properties. Therefore, C-PC was presented as a possible chemotherapeutic agent (Deniz et al., 2016; Mahendran et al., 2022; Deniz et al., 2016; Katari et al., 2023). In a prior study, the inhibitory effects of C-PC generated from *Spirulina platensis* on human ovarian cancer cells (SKOV-3) revealed that, after 24 and 48 h of C-PC treatment, the IC50 values were 216.6 μM and 163.8 μM, respectively (Ying et al., 2016). Another investigation assessed the impact of phycobiliprotein extract from *Arthrospira platensis* on four cancer cell lines: glioblastoma (295SF), prostate cancer (3PC), colorectal cancer (116HCT), and leukemic cancer cells (60HL). The most noteworthy outcomes were displayed by leukemia cancer cells, with an IC50 of 112.6 μg/mL (Viana Carlos et al., 2021). In a prior study, supercritical fluid extraction (SCF) of phycocyanin (PC) from Spirulina platensis were tested against lung cancer cell line (A549) to determine cytotoxicity. SCF extract had an IC50 value of 26.82 μg/ml (Deniz et al., 2016). Lung cancer is the second most frequent cancer globally and has the highest death rate for both men and women (Miller et al., 2016). Based on histology, lung cancer can be classified into two main categories: small-cell lung cancer (SCLC) and non-small-cell lung cancer (NSCLC). Calu-6 is a human non-small cancer cell line that is derived from lung adenocarcinoma (Tripathi et al., 2019). In this regard, the anticancer effects of C-PC, isolated from *Cyanobium* sp. MMK01, on human lung cancer cells (Calu-6), was assessed through the MTT assay. Following a 24- and 48-h PC treatment, the IC50 values were found to be 20 μg/mL and 15 μg/mL, respectively. These results were considerably less than those previously documented (Figure 5A). Furthermore, fluorescence microscopic visualization of cell staining with FDA/PI confirmed the results of the MTT assay, which showed a significant decrease in the ratio of live to dead cells of Calu-6 treated with the IC50 concentration of C-PC. The type, chemical structure, and purity level of C-PC from various cyanobacterial strains, as well as the varying sensitivity of different types of cells to C-PC, would be contributing factors for the discrepancy between the IC50 of our investigation and other studies.

Human diseases, including cancer and cardiovascular disorders, appear to be affected by free radicals, especially when their production increases (Deighton et al., 2000). C-PC is a promising therapeutic alternative for many disorders because it has significant radical scavenging activity (Hernández Martínez et al., 2023). In a previous study, phycocyanin extracted from *Lyngbya* sp. A09DM showed 50% DPPH inhibitory efficacy at a concentration of 273.31 μg/mL (Sonani et al., 2014). *Pseudanabaena* sp., *Limnothrix* sp., and *A. platensis* were among the filamentous cyanobacteria whose antioxidant activity was investigated by Aoki et al. (2021). Pure C-PC demonstrated 84% maximal radical-scavenging activity at 1 mg mL^−1^ (Aoki et al., 2021). Nevertheless, the pure C-PC derived from Cyanobium sp. MMK01 exhibited a 48% suppression of DPPH radicals at a concentration as low as 200 μg/mL. Gas-chromatography mass spectrometry (GC–MS) analysis of methanolic MMK01 cell extract revealed the presence of fatty acids, alcohols like phytol, phthalates, and hydrocarbons with antioxidant, anti-inflammatory, antibacterial, and anti-cancer properties, which could be a reliable source of biologically active compounds. The main compound found in the methanolic extract of cyanobacterium *A. platensis* was heptadecane, which is consistent with the findings of our study (Kumar et al., 2011; Chagas et al., 2021; Gheda et al., 2023). Nevertheless, more investigation is required to picture cyanobacterial crude extracts as an inexpensive, natural, and safe source for pharmaceutical usage after thorough clinical trials.

## 5 Conclusion

This work is the first investigation into the simple and affordable extraction and purification of C-PC from the unique cyanobacterium, *Cyanobium* sp. MMK01, where the obtained C-PC reached the exceptional purity index of 5.82. The current research is highly significant from two perspectives: first, the discovery and identification of a new strain of cyanobacteria with extraordinary potential to produce C-PC that can rival the strains offered for this pigment’s production, and second, it opens an avenue for the development of a straightforward and affordable method for C-PC pigment extraction and purification. Therefore, based on all the data provided in this study, the MMK01 strain has a great deal of potential for producing C-PC that may replace *Arthrospira* species, showing promise as a possible therapeutic agent. More study is required to pinpoint putative strains in the cyanobacteria phylum that produce significant amounts of C-PC, establish substitute purification methods, look into the mechanisms underlying the bioactivities of C-PC, and assess its effectiveness *in vivo*.

## Data Availability

The datasets presented in this study can be found in online repositories. The names of the repository/repositories and accession number(s) can be found in the article/Supplementary material.
