# Supplementary figures and images for: A new and promising C-phycocyanin-producing cyanobacterial strain, Cyanobium sp. MMK01: practical strategy towards developing a methodology to achieve C-phycocyanin with ultra-high purity
Source: Front Microbiol. 2024 Nov 21;15:1394617. doi: 10.3389/fmicb.2024.1394617 (PMC11617521; doi:10.3389/fmicb.2024.1394617)

Supplementary material

**
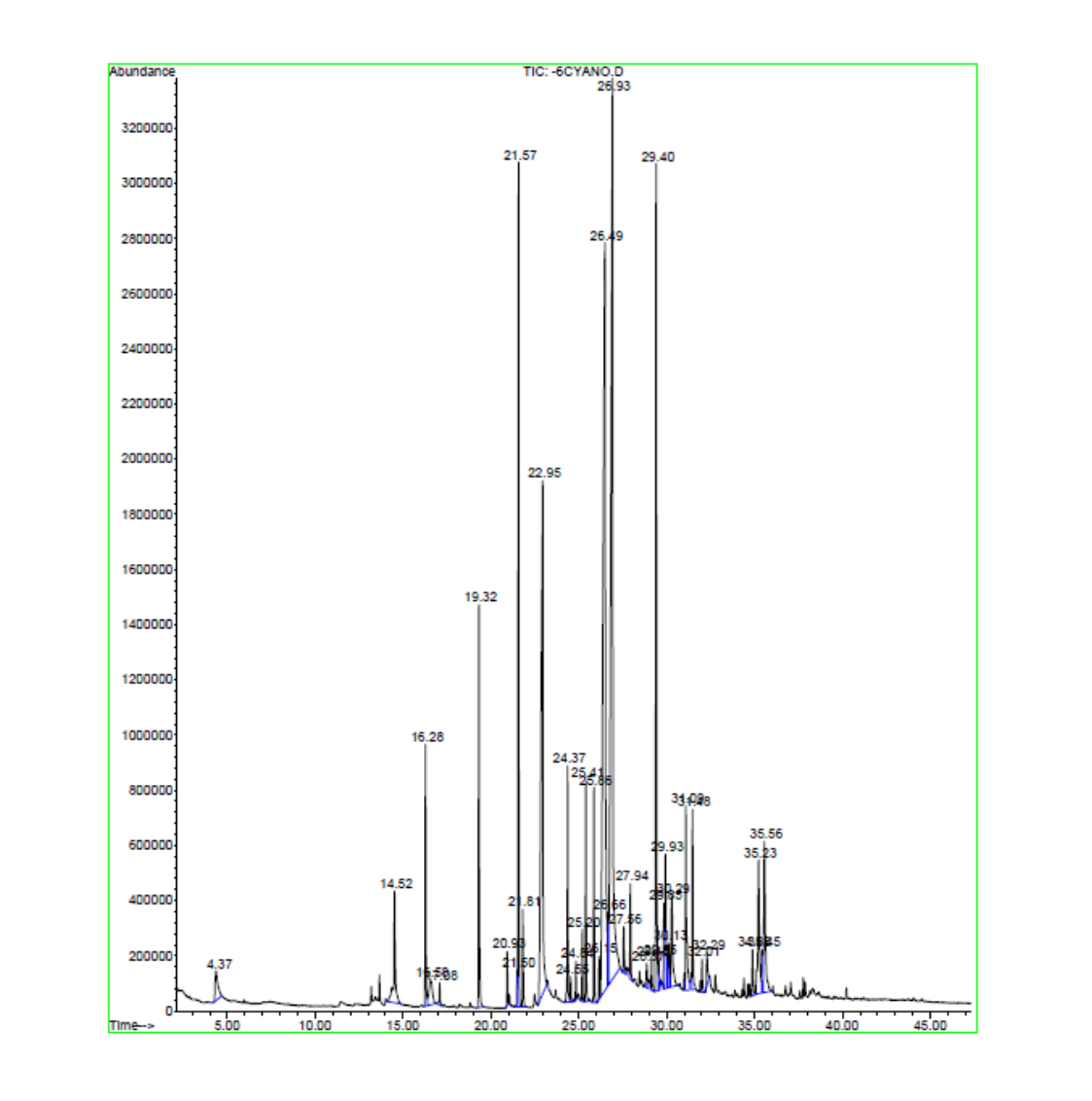
Fig. S1.** GC-MS analysis of *Cyanobium* sp. MMK01 cell extract.

Supplement: Supplementary file 1 [file Supplementary_file_1.docx]
